# The Efficacy of the Ponseti Method in the Management of Clubfoot: A Systematic Review

**DOI:** 10.7759/cureus.52482

**Published:** 2024-01-18

**Authors:** Hassan B Maghfuri, Ali A Alshareef

**Affiliations:** 1 Orthopedic Surgery, Ministry of Health, Jizan, SAU; 2 Pediatric Orthopedics, Abha Maternity and Children Hospital, Abha, SAU; 3 Orthopedic Surgery, Armed Forces Hospital Southern Region, Khamis Mushait, SAU

**Keywords:** foot deformity, clubfoot, ponseti, rate, success

## Abstract

Clubfoot is a congenital abnormality of the lower extremities, and it may be unilateral or bilateral. Left untreated, it may lead to issues with walking. Additionally, inappropriate treatment or the lack of treatment can result in functional damage. The goal of clubfoot treatment is to correct the deformities of the involved components. The Ponseti method has been regarded as the gold standard for the treatment of clubfoot as it is safe and effective. In this review, we aimed to assess the success of the Ponseti method in the treatment of clubfoot by reviewing the previous studies on this subject. We searched electronic databases, including PubMed, Scopus, Science Direct, and Google Scholar, for relevant articles spanning the period from 2018 to 2023. The keywords used in the search were "Ponseti method, Treatment, Outcomes, Success, Relapse, Failure, and Rates." The inclusion criteria were original articles in English on clubfoot patients treated with the Ponseti method.

While our search yielded a total of 1,037 articles, only nine were deemed eligible for analysis based on the inclusion criteria. The articles involved a total of 537 feet of 358 patients and the age of the patients ranged from one day to five years. The success rate ranged between 55% and 100%, and the relapse rate ranged between 3.2% and 34.2%. Based on our findings, the Ponseti method has a high success rate in the treatment of idiopathic clubfoot, and hence it is an excellent conservative method of treatment. However, there are additional factors that may affect the treatment outcomes, which need to be taken into account.

## Introduction and background

Clubfoot is a congenital deformity of the lower extremities [1]. This condition affects the musculoskeletal structures of the foot, including the equinus, varus, cavus, and adducts [2]. The incidence of clubfoot is one to two cases per 1000 live births, and it is three times more prevalent among males compared to females. Clubfoot is present unilaterally or bilaterally in 50% of cases [2-8]. The goal of clubfoot treatment involves correcting the deformities of the involved components through gradual ligamentous and muscular lengthening to attain a flexible plantigrade foot with no pain [3,4]. Inappropriate treatment or the lack of treatment can result in functional damage, leading to changes in the bone structures [7,8]. Untreated individuals with clubfoot suffer from issues with walking as they walk on the sides of their feet [9,10].

The treatment of clubfoot can be challenging due to the pathological anatomy of the foot [11,12]. Several interventions have been put forward, including surgical methods [12]. However, in the mid-1940s, a Spanish orthopedic doctor named Ignacio Ponseti conducted several in-depth studies on the pathological and functional anatomy of the clubfoot, eventually perfecting what came to be known as the Ponseti method [5,6,8]. The Ponseti method has been considered the gold standard for the treatment of clubfoot as it is safe and effective. It is based on gentle and repetitive manipulations to stretch the soft tissues progressively, followed by weekly cast immobilization [1]. This systematic review explored various published articles on the topic to assess the success rates and relapse rates in patients treated for clubfoot with the Ponseti method.

## Review

Methodology

Study Design and Search Strategy

We adhered to the Preferred Reporting Items for Systematic Reviews and Meta-Analysis (PRISMA) checklist [13] to perform this systematic review. Electronic databases, including PubMed, Scopus, Science Direct, and Google Scholar, were searched to obtain relevant articles. The search process was limited to articles published between 2018 and 2023. A set of keywords was used for searching purposes, including "Ponseti method, Treatment, Outcomes, Success, Relapse, Failure, and Rates;" these keywords were used in different combinations to obtain the maximum possible related articles. The obtained titles were revised to exclude irrelevant articles, such as those reporting complex clubfoot or clubfoot with associated etiology and studies that reported combination interventions with the Ponseti method.

Eligibility Criteria

**Figure 1:**
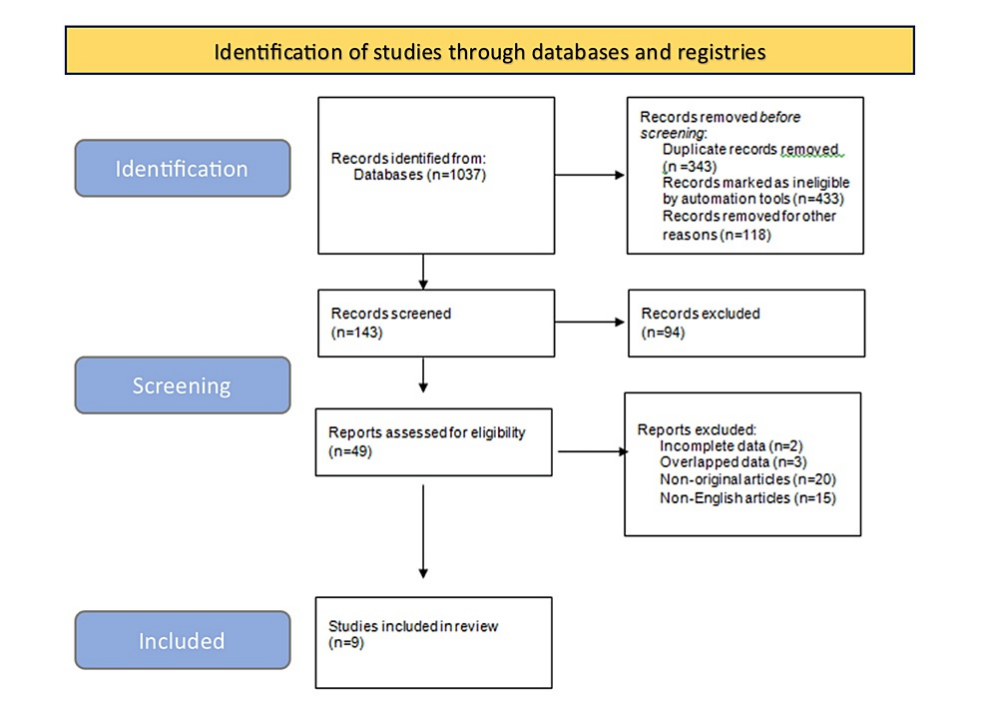
Search strategy and selection of articles

The articles related to the current subject were checked thoroughly, and duplicates were excluded. The remaining articles were then examined to choose only original research articles reporting the outcomes of the Ponseti method, whereas systematic reviews, letters to the editor, reviews, and meta-analyses were excluded. Only articles in English were defined as relevant, which were then included in the second stage. The second step involved manually reviewing the abstracts to select the relevant studies for revision. The inclusion criteria were as follows: studies of any design conducted on children of any age. Articles with only abstracts available were excluded, and articles reporting overlapping or incomplete data were also excluded. The search strategy and selection of articles are depicted in Figure 1.

Data Review and Analysis

Data review included a preliminary review of the abstracts of the studies to determine the data of interest for extraction. Then, the full-text articles were reviewed. A specially designed Excel sheet was used for data extraction and revision. A pre-designed table was used for summarizing the extracted data.

Results

This systematic review included nine studies that met the inclusion criteria [14-22] (Table 1). Two studies did not report the study design [21,22]. While most of the studies were prospective [15,16,17,18], one was descriptive-retrospective [14], one was retrospective [19], and one employed a cross-sectional design [20]. These studies involved a total of 358 patients 537 feet. While one study explored three types of clubfoot - idiopathic, neurogenic, and syndromic [14] - the remaining eight studies were on idiopathic clubfoot. The age of patients ranged from one day to five years. Two studies categorized patients into two groups based on their age; the younger group, which involved children aged less than two years [18] or infants aged less than 30 days [19]; and the older group, which included children aged two to five years [18] or infants who started treatment after 30 days [19]. The overall success rate was reported in eight studies, whereas only one study categorized the success rate [22]. The overall success rate ranged between 55% [18] and 100% [15,19]. One study reported that the success rate was the lowest (55%) among older children aged two to five years [18]. The study that categorized the success rate reported excellent outcomes in 64.86% of the subjects, good outcomes in 29.72%, and poor outcomes in 5.4% [22]. Only three studies [15,18,22] reported the duration of follow-up during which outcomes were determined, and the follow-up duration ranged between 6 and 18 months.

**Table 1:** Summary of the included studies CTEV: congenital talipes equinovarus

Author and year of publication	Study design	Sample size, type of clubfoot, and age	Success and relapse rates	Results and main findings
Alhunaishel et al., 2023 [14]	Descriptive-retrospective	N=93 patients (136 feet). Type: idiopathic: 71 (76.3%), neurogenic: 14 (15.1%), syndromic: 8 (8.6%). Age: 1D-5Y	The initial success rate was 97.9%; 4.4% relapsed but was treated with repeated Ponseti method	Patients with congenital clubfoot were successfully managed by the Ponseti protocol with a high success rate
Mishra and Muthaiyan, 2023 [15]	Prospective	N=30 patients (40 feet). Type: idiopathic. Age: 7D-2Y	The success rate was 90% after 18 months (excellent to good outcomes). The relapse rate was 10% (poor outcomes)	Patients presenting <7 months showed excellent results. Poor results (relapse) were seen in non-compliant patients. The Ponseti method is an excellent conservative method for the treatment of congenital clubfoot
Maleki et al., 2022 [16]	Prospective-observational	N=27 patients (38 feet). Type: idiopathic. Age: 1-73D	The success rate was 100%; all patients achieved complete correction. The relapse rate was 34.2%	The mean age of relapsed cases was more than those without relapses. Cases with a higher initial Dimeglino score had a higher recurrence rate. The treatment should be initiated as soon as possible as it is more effective at a younger age. Severe cases at the initial visit had a higher recurrence rate
Mousa et al., 2021 [17]	Prospective	N=26 patients (40 feet). Type: idiopathic. Age: 1W-1.5Y	The success rate was 95%	The final results of correction according to the Pirani score were excellent in 18 feet (45%), fair in 20 feet (50%), and poor in two feet (5%). The Ponseti technique of serial manipulation and cast is an easy, efficient, economical, and reliable method of CTEV correction when it is applied early
Ahmed et al., 2021 [18]	Prospective	N=40 patients. Type: idiopathic. Age: ˂2-5Y. Group A: ˂2Y (20 patients). Group B: 2-5Y (20 patients)	The success rate was 85% in Group A and 55% in Group B after six months	Patients aged <2 years have significantly higher success rates compared to patients aged between two and five years. The Ponseti method is recommended as standard therapy in clubfoot management for patients aged <2 years and for correction of mild and moderate deformities in patients aged two to five years
Lee et al., 2020 [19]	Retrospective	N=54 patients (77 feet). Type: idiopathic. Age: ˂30D-˃30D (1 month). Group A: 21 started treatment before 30D. Group B: 33 started treatment after 30D	The success rate was 100%. Relapse was 3.2% in Group A and 6.5% in Group B	The difference in the rate of relapse between the two groups was not statistically significant (p=0.6). All of the relapsed feet were successfully treated with the repeated Ponseti method. Treating CTEV using the Ponseti method starting after one month was not associated with a higher rate of relapse
Ahmad et al., 2020 [20]	Cross-sectional	N=58 patients (88 feet). Type: idiopathic. Age: 2-12 M	The success rate was 91% (excellent outcome)	9% of patients left treatment and lost to follow-up. Patients with early presentation and low pretreatment Pirani scores showed excellent outcomes compared to patients with high Pirani scores. The Ponseti approach led to excellent outcomes for the majority of patients
Shinde et al., 2018 [21]	------	N=30 patients (48 feet). Type: idiopathic. Age: 1-24W	The success rate was 89.58%. The relapse rate was 20.83%	79.16% of feet had an excellent outcome, 10.42% had a fair outcome, and 10.42% had a poor outcome. There were seven (14.58%) patients with cast-related complications. The Ponseti method is a reproducible, safe, and effective intervention for CTEV correction, reducing the rate of extensive corrective surgeries for clubfoot correction
Sankar Mohan et al., 2018 [22]	-------	N=30 patients (30 feet). Type: idiopathic. Age: ˂1M-˃6M	The success rate at six months: 64.86% excellent, 29.72% good, and 5.4% poor	The Ponseti method is a cost-effective modality for congenital idiopathic clubfoot, resulting in excellent outcomes and radically reducing the need for extensive corrective surgery

The rate of relapse was not reported in three studies [17,18,20], and one study reported poor outcomes but did not touch on relapse [22]. The relapse rate ranged between 3.2% [19] and 34.2% [15]. Furthermore, one study reported that the relapse rate was higher among infants who started treatment after 30 days of age [19]. Another study revealed that the relapsed children (4.4%) were retreated with the Ponseti method [14]. Good and excellent outcomes from the Ponseti method were reported among patients of younger age with an early presentation [15,20], those aged less than two years [18], and those with low Pirani scores [20]. Also, it was reported that the method was more effective in patients of younger ages [16]. Poor outcomes and relapse after Ponseti were reported in non-compliant patients [15], those older, and those with higher initial Dimeglino scores [16]. However, one study reported no significant difference in relapse rate between those who were treated before 30 days and those treated after 30 days in age [19]. Only one study reported cast-related complications, at a rate of 14.58% [21].

Discussion

Clubfoot, also known as congenital talipes equinovarus (CTEV), is a congenital abnormality of the foot [12]. The etiology of clubfoot may be associated with arthrogryposis, myelodysplasia, or multiple congenital deformities, but the most common presentation involves clubfoot in isolation, which is considered the idiopathic type [2]. In the current review, we included studies on idiopathic clubfoot in isolation treated with the Ponseti method to determine its success and relapse rates.

The success rate as reported in the included studies ranged between 55% and 100%; however, six studies reported a success rate of over 90% [14,15,16,17,19,20]. Furthermore, of these six studies, two studies reported a 100% success rate [16,19]. The rate of relapse ranged between 3.2% and 34.2%. Based on these findings, the success rate of the Ponseti method as a conservative intervention for clubfoot is very high. A previous report showed that the initial correction rate of clubfoot ranges between 93% and 100%, whereas the relapse rate is between 14% and 48% [23]. However, the relapse rates in our studies were less than those reported in that study [23]. In a systematic review involving 11 studies with a total of 374 non-idiopathic and 801 idiopathic clubfeet, non-idiopathic clubfeet were found to have a higher recurrence rate of 43.3% compared to 11.5% of recurrent idiopathic cases with a lower rate of success rate (69.3% vs. 95%, respectively) [24].

In our analysis, only one study included other types of clubfoot, including syndromic and neurogenic; however, that study did not compare the success rates of each type. However, it was reported that even in relapsed patients, the Ponseti method was used for the successful re-treatment of the patients [14]. Therefore, it is necessary to compare the success rate of the Ponseti method regarding different types of clubfoot. In a systematic review of relapse involving 10,500 feet from 84 studies, it was found that the outcomes varied significantly; the rate of recurrence ranged from 1.9% and 45% [25]. These rates of relapse are much higher compared to ours, where the relapse rates ranged between 3.2% and 34.2% only. Another study involving 240 feet with idiopathic clubfoot of 200 patients reported a relapse rate of 6.25% (15 feet) after treatment with the Ponseti method [26]. The rate of relapse can also be indicative of success rates, where low rates of relapse translate into higher rates of success of the method.

The success rates and relapse rates were noted to vary greatly between studies, and this may be attributed to various additional factors. It was demonstrated that the success of the Ponseti method depends on age, gender, and early diagnosis [1]. Conservative treatment in the first weeks of life has been recommended for clubfoot [3,8,11,27]. However, it was shown that the age at which treatment begins makes no significant difference, and a clubfoot can be corrected later on [28]. In our analysis, we found that children who presented and were treated at younger ages tended to report good and excellent outcomes from the treatment at a higher rate. However, none of the studies assessed the correlation between the age at the time of treatment and the treatment outcomes. On the other hand, those with poor outcomes tended to be older; however, there was no agreement between the studies as to the exact age above which poor outcomes are more likely or the optimum age range at treatment to obtain excellent outcomes.

In a previous study, the correlation between the efficacy of the Ponseti method for treating clubfoot and the age of treatment onset was investigated. The study categorized patients into two groups; the first group included patients who underwent treatment early with a mean age of two months and seven days, whereas the second group included children treated much later at a mean age of 17 months and 13 days. A significant correlation was reported between the effectiveness of the method and the age of treatment onset. Additionally, it was reported that the ideal age for the treatment of clubfoot was 0-6 months, especially to avoid relapse [12]. However, in our analysis, infants aged less than two years reported a high success rate of 85%, but the relapse rate was not reported. Therefore, the optimum age to start treatment is still a matter of uncertainty.

A systematic review assessed the management of clubfoot of infants under two years of age included 12 articles that showed the effectiveness of the Pinseti method; however, relapse was reported in nine studies with a maximum relapse rate of 27.1% due to non-adherence with bracing and other factors such as low income and social and economic status [29]. Also, non-compliance was one of the factors found to be common among those who reported such outcomes, but only one study reported such a factor [15]. A previous study involving 240 feet with idiopathic clubfoot also reported that patients who experienced recurrence before the age of two years tended to be significantly more non-adherent with bracing [26].

The Pirani classification system is a reproducible and valid approach for the evaluation of clubfoot; it is used for the measurement of the severity of clubfoot before the initiation of treatment as well as at the end of the treatment [3,5,7,30]. The score ranges from 0 to 6, where 0 refers to normal feet, and 6 stands for the most severe deformity [31]. The Dimeglio classification can also be used to assess the clubfoot; it is based on the flexibility of the forefoot and hindfoot and classifies feet into four types. Type I refers to flexible hindfoot and forefoot, type II indicates rigid hindfoot and flexible forefoot, in type III, only the forefoot is rigid, and in type IV, both the hindfoot and forefoot are rigid [6]. It has a maximum score of 20 points, and the deformity is graded as benign, moderate, severe, and very severe [32]. In our analysis, patients with poor outcomes had higher Dimeglino scores before treatment, and those with good and excellent outcomes had low Pirani scores. One systematic review has reported that few studies followed patients for more than five years [25]. In our analysis, only three studies reported the duration of follow-up of patients, and it was generally short, ranging from six to 12 months [15,18,22].

## Conclusions

The Ponseti method is an excellent conservative method for the treatment of idiopathic clubfoot with high success rates and low relapse rates. Excellent outcomes can be obtained in younger children, with low Pirani or Dimeglino scores, and adhere to the treatment protocol, with good to fair results reported in older patients or in those who have higher Pirani or Dimeglino scores, or at least a decrease in the need for extensive surgical release. The optimum age for initiating treatment is not well defined; however, the treatment should be initiated promptly to attain excellent outcomes.
